# Vaccination against SARSCoV-19 among psychiatric patients at the central Greek hospital

**DOI:** 10.1192/j.eurpsy.2023.1682

**Published:** 2023-07-19

**Authors:** M. E. Anagnostaki, I. Anagnostaki, G.-E. Papaspiropouloou

**Affiliations:** Internal Medicine Dpt, Psychiatric Hospital of Attika, Athens, Greece

## Abstract

**Introduction:**

Vaccination against SARSCov-19 all over Europe reached over 80% of adult population confronting the pandemic burden on National Health Systems. On the contrary large parts of population remained unvaccinated. These groups are mainly individuals with poor socioeconomic status and psychiatric patients

**Objectives:**

to determine the ratio of fully vaccinated patients among the hospitalized and outpatient of Psychiatric Hospital of Attika. The reason of vaccination avoidance recorded by the clinician

**Methods:**

The study has done retrospectively and included 2583 psychiatric patients who are hospitalized or are visiting the Outpatient clinic. A concise questionnaire was formed to record the main reason of avoidance (Denial/Medical Issues/ Loss of follow up/ other)

**Results:**

520 out of 2583 (21%) remained not fully vaccinated throughout the pandemic and denial by the patient was the main reason (55%). The reasons recorded at the patient’s file by the physician are shown at table 1.

**Table 1:** main reasons of vaccine avoidance.

Denial	55%
Medical contraindications	15%
Loss of follow up	26%
other	4%

**Conclusions:**

Psychiatric patients belong to a high probability group for vaccine avoidance. In our study the frequency of unvaccinated psychiatric patients found greater than healthy population and the main reason is patient decision not to consent. Loss of information, distrust, inadequate social help are causes of poor decision making and consequent low quality health services

**Disclosure of Interest:**

None Declared

